# Supplementary material for: Baseline Characterization of the Gut Microbiota of Field and Colony Populations of Phlebotomus tobbi and Preliminary Assessment of the Anti-Leishmanial Activity of Cultivable Bacteria
Source: Pathogens. 2026 Jun 23;15(7):658. doi: 10.3390/pathogens15070658 (PMC13416292; doi:10.3390/pathogens15070658)
Supplement: Supplementary file 1 [file pathogens-15-00658-s001.zip › pathogens-4357168-File S1/Taxa2ASV_Decomposer/Family/f__Enterobacteriaceae/heatmap_with_clustering.pdf]

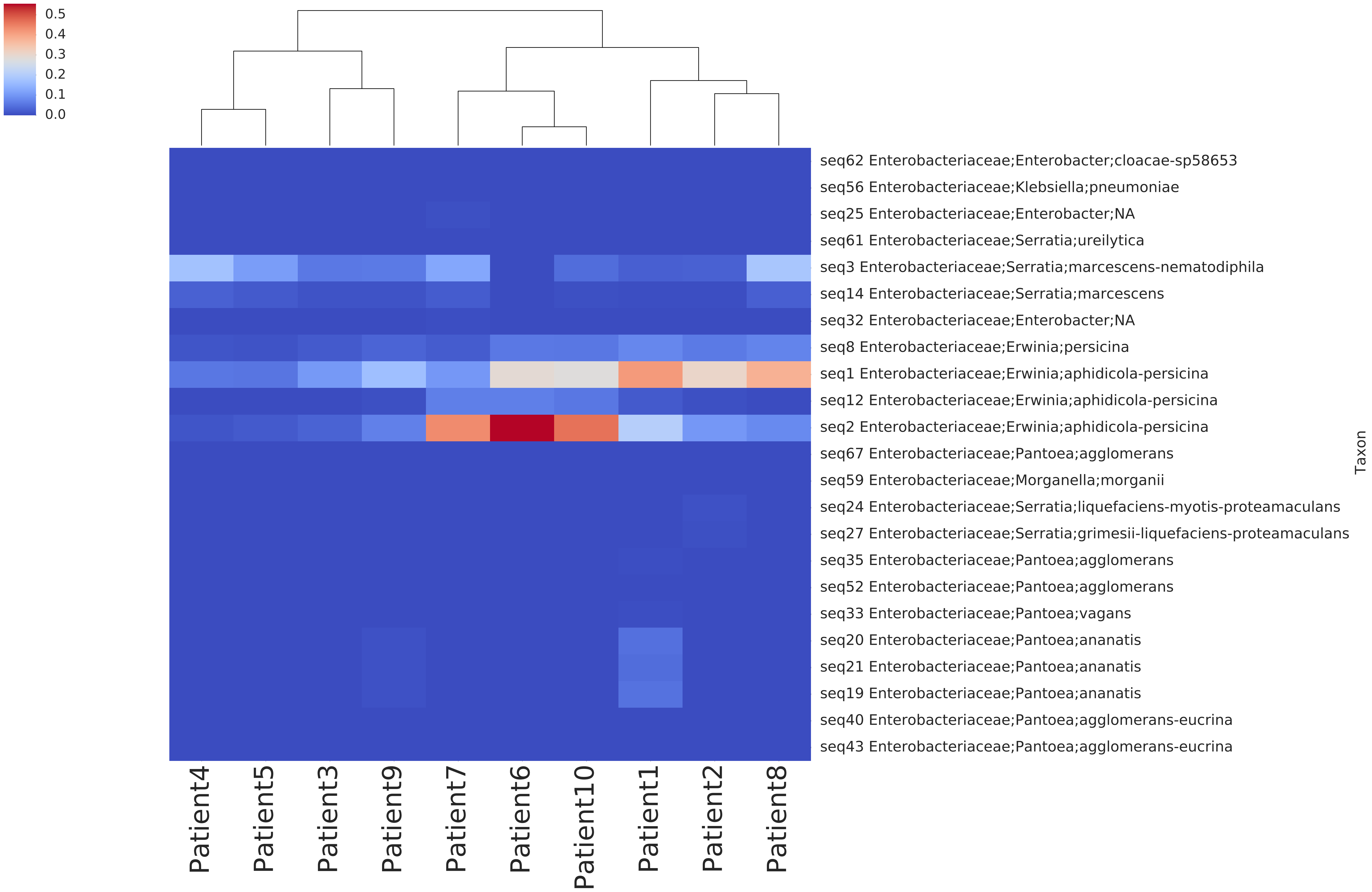

0.5
0.4
0.3
0.2
0.1
0.0
seq62 Enterobacteriaceae;Enterobacter;cloacae-sp58653
seq56 Enterobacteriaceae;Klebsiella;pneumoniae
seq25 Enterobacteriaceae;Enterobacter;NA
seq61 Enterobacteriaceae;Serratia;ureilytica
seq3 Enterobacteriaceae;Serratia;marcescens-nematodiphila
seq14 Enterobacteriaceae;Serratia;marcescens
seq32 Enterobacteriaceae;Enterobacter;NA
seq8 Enterobacteriaceae;Erwinia;persicina
seq1 Enterobacteriaceae;Erwinia;aphidicola-persicina
seq12 Enterobacteriaceae;Erwinia;aphidicola-persicina
seq2 Enterobacteriaceae;Erwinia;aphidicola-persicina
seq67 Enterobacteriaceae;Pantoea;agglomerans
seq59 Enterobacteriaceae;Morganella;morganii
seq24 Enterobacteriaceae;Serratia;liquefaciens-myotis-proteamaculans
seq27 Enterobacteriaceae;Serratia;grimesii-liquefaciens-proteamaculans
seq35 Enterobacteriaceae;Pantoea;agglomerans
seq52 Enterobacteriaceae;Pantoea;agglomerans
seq33 Enterobacteriaceae;Pantoea;vagans
seq20 Enterobacteriaceae;Pantoea;ananatis
seq21 Enterobacteriaceae;Pantoea;ananatis
seq19 Enterobacteriaceae;Pantoea;ananatis
seq40 Enterobacteriaceae;Pantoea;agglomerans-eucrina
seq43 Enterobacteriaceae;Pantoea;agglomerans-eucrina
Taxon
Patient4
Patient5
Patient3
Patient9
Patient7
Patient6
Patient10
Patient1
Patient2
Patient8
